# Workplace Stressors and Work Engagement Among Married Nurses: The Roles of Psychological Distress and Perceived Partner Responsiveness

**DOI:** 10.3390/healthcare14172746

**Published:** 2026-08-28

**Authors:** Hu Ying, Jie Liu, Xiaofu Pan

**Affiliations:** College of State Governance, Southwest University, Chongqing 400715, China; inmhoo@email.swu.edu.cn (H.Y.); jackliu@email.swu.edu.cn (J.L.)

**Keywords:** organizational dehumanization, patient/family incivility, psychological distress, work engagement, perceived partner responsiveness, nurses

## Abstract

**Highlights:**

**What are the main findings?**

- Organizational dehumanization and patient/family incivility were independently associated with greater psychological distress and, indirectly, with lower work engagement.
- Both the distress–engagement association and the corresponding indirect associations were weaker at higher levels of perceived partner responsiveness.

**What are the implications of the main findings?**

- Nursing workforce mental health strategies should address both dehumanizing organizational practices and patient/family incivility.
- Family-domain relational resources may complement, but should not replace, organizational efforts to reduce workplace strain and support recovery.

**Abstract:**

**Background/Objectives:** Understanding threats to nurses’ work engagement is important for workforce stability, care quality, and nurses’ well-being. We examined whether organizational dehumanization and patient/family incivility were indirectly associated with lower work engagement through psychological distress and whether perceived partner responsiveness moderated the distress–engagement association. **Methods:** Three-wave survey data were collected from 325 married nurses at one-week intervals. Workplace stressors were measured at Time 1, psychological distress and perceived partner responsiveness at Time 2, and work engagement at Time 3. Covariate-adjusted moderated mediation analyses were conducted using 5000 bootstrap samples. **Results:** Organizational dehumanization (*β* = 0.438, *p* < 0.001) and patient/family incivility (*β* = 0.271, *p* < 0.001) were independently associated with greater psychological distress, which was associated with lower work engagement (*β* = −0.246, *p* < 0.001). The psychological distress × perceived partner responsiveness interaction was significant (*β* = 0.305, *p* < 0.001), explaining an additional 6.3% of the variance in work engagement. The negative distress–engagement association was significant at low and mean levels of perceived partner responsiveness but not at a high level. Both workplace stressors showed significant indirect associations with work engagement at low and mean, but not high, levels of perceived partner responsiveness; the corresponding high–low contrasts were significant. **Conclusions:** The findings are consistent with a pattern in which workplace stressors from different sources are associated with lower work engagement through psychological distress, while higher perceived partner responsiveness is associated with a weaker distress–engagement relationship. Reducing avoidable workplace stressors and supporting recovery outside work may help sustain nurses’ engagement; causal and intervention effects remain to be tested.

## 1. Introduction

High-quality nursing care depends on nurses remaining psychologically present and engaged despite the emotional demands of care work. Work engagement reflects the physical, emotional, and cognitive energy nurses invest in their roles [1]. In nursing, work engagement has been linked to favorable personal and professional outcomes and care performance [2]. Systematic-review evidence also links healthcare staff engagement to patient safety [3] and work engagement to quality of care [4]. In healthcare systems facing persistent workforce strain, identifying avoidable workplace stressors and resources that support recovery is important for both nurses and care delivery. This requires looking beyond workload and staffing to consider how nurses are treated by their organizations and by the patients and family members with whom they interact.

Organizational dehumanization occurs when employees perceive that their organization treats them primarily as instruments for achieving its goals rather than as people with dignity, needs, and agency [5]. Research has examined procedural justice as an antecedent of organizational powerlessness and dehumanization [6] and has linked organizational dehumanization to emotional labor and poorer job attitudes [7,8], psychological need thwarting and strain-related outcomes [9,10], and deviant behavior among nurses [11]. Studies of office design and leader–member exchange further indicate that organizational arrangements and supervisors can shape employees’ sense of being dehumanized [12,13], while cross-cultural evidence suggests that the strength of its consequences may vary across national contexts [14]. In nursing, organizational dehumanization has been associated with lower work engagement through work stress [15] and with professional practice environments and work passion [16]. This evidence establishes its relevance but leaves two gaps: most studies examine organizational dehumanization separately from mistreatment by service recipients, and few test whether a resource outside work conditions its downstream association with engagement.

Strain may also arise at the point of care, where nurses interact directly with patients and their families. Patient/family incivility refers to low-intensity rude, discourteous, or disrespectful behavior whose intent to harm is ambiguous [17]. The wider incivility literature shows that even low-intensity disrespect is associated with adverse employee outcomes [18], while research on customer aggression and outsider verbal abuse links these experiences to emotion regulation and emotional exhaustion among service employees [19,20]. In nursing, patient/family incivility has been linked to burnout [21], and experimental evidence indicates that rudeness can impair medical-team performance [22]. Incivility should not be equated with overt violence. However, systematic reviews and nursing studies show that aggression and violence from patients and visitors are consequential occupational exposures [23,24,25], underscoring the broader importance of mistreatment originating from care recipients. Thus, patient/family incivility warrants examination as a distinct interpersonal demand rather than as a minor inconvenience or a proxy for organizational treatment.

Although both stressors convey disregard for nurses, organizational dehumanization and patient/family incivility differ in source and form. Organizational dehumanization is a generalized perception that the organization treats employees as interchangeable means to institutional ends, denying their individuality and agency [5]. Patient/family incivility, by contrast, comprises discrete episodes of discourteous or disrespectful conduct by patients or family members during care delivery [21]. The former is embedded in the employee–organization relationship and signals organization-based devaluation; the latter arises at the care interface and constitutes an immediate interpersonal demand. Because the two stressors may co-occur, examining them simultaneously allows their unique associations with psychological distress to be estimated. It also tests whether demands from different sources converge on a common strain response without assuming identical proximal processes.

Job demands–resources (JD–R) theory and conservation of resources (COR) theory explain why these demands may generate strain. JD–R theory identifies a health-impairment process in which sustained job demands consume cognitive and emotional effort [26,27]. COR theory adds a resource-loss account: instrumental or disrespectful treatment may threaten valued resources, including emotional energy and control, and increase vulnerability to further loss [28,29]. However, these broad frameworks do not by themselves specify whether demands from different social sources retain independent associations with strain or where a resource outside work enters the process. The work–home resources model addresses this cross-domain issue by explaining how contextual resources in the home domain can generate personal resources that support subsequent functioning at work [30]. Work–family enrichment theory likewise proposes that resources acquired in one role can improve functioning in another [31], but the work–home resources model more directly identifies the resource-transfer process and is therefore used here to position partner responsiveness at the later distress–engagement stage.

Psychological distress may be the common proximal response through which these distinct demands are associated with lower work engagement. The routes to distress may differ: organizational dehumanization may erode nurses’ dignity, professional worth, and agency, whereas patient/family incivility may create an immediate need to regulate negative emotions during care. Emotional-labor theory identifies emotion regulation itself as a source of work strain [32], and longitudinal person-centered research shows that employees can follow distinct, relatively persistent emotional-labor profiles [33]. Research linking workplace mistreatment to emotional-labor profiles further supports emotion regulation as one route from adverse treatment to strain [34]. Existing nursing studies nevertheless stop short of the integrated pathway tested here: organizational dehumanization has been examined in relation to work stress and engagement [15], whereas patient/family incivility has been examined in relation to burnout [21]. Neither general work stress nor burnout is equivalent to psychological distress, and neither study considered both stressors as antecedents of the same strain response. Psychological distress may therefore capture a shared loss of attentional and emotional resources needed for sustained work involvement.

Resources outside work may not prevent psychological distress, but they may shape its association with subsequent work engagement. Perceived partner responsiveness refers to the extent to which individuals feel understood, validated, and cared for by an intimate partner [35]. Unlike general social or family support, which may be provided through multiple relationships, perceived partner responsiveness concerns the perceived quality of a specific partner’s response. It is also distinct from marital satisfaction, which reflects a global evaluation of the relationship. Ten-year longitudinal evidence showed that perceived partner responsiveness predicted increases in eudaimonic—but not hedonic—well-being and that lower affective reactivity to daily stressors partly accounted for this longitudinal association [36]. These findings point to a stress-regulatory role for partner responsiveness, but whether that role extends to work engagement remains untested. Under the work–home resources model, perceived partner responsiveness can be conceptualized as a home-domain relational resource that may help preserve or replenish personal resources available for work [30]. Perceived partner responsiveness is therefore positioned at the distress–engagement stage. This placement does not imply that responsiveness is unrelated to distress; rather, the model tests whether higher responsiveness is associated with a weaker distress–engagement relationship and, consequently, weaker indirect associations between both workplace stressors and engagement through distress.

For married nurses, a spouse is a recurring figure in the home domain who may help them process difficult care experiences and regulate emotions after demanding shifts. Whether these interactions are restorative, however, depends less on marital status than on whether the spouse is perceived as understanding, validating, and caring [35,36]. The present study therefore treats marriage as the relational context and perceived partner responsiveness as the resource. Restricting the sample to married nurses provided a clearly identified spousal referent and allowed the analysis to focus on differences in responsiveness within that context; it did not test whether married nurses fare better than unmarried nurses. The proposed mechanism may also operate among nurses in cohabiting or other committed relationships. More generally, social support can buffer the effects of stress [37], while JD–R theory identifies coworker and supervisor support as potential job resources [27].

Taken together, the proposed model distinguishes workplace demands by source, examines psychological distress as a shared strain pathway, and locates perceived partner responsiveness as a home-domain resource at the distress–engagement stage.

**Figure 1:**
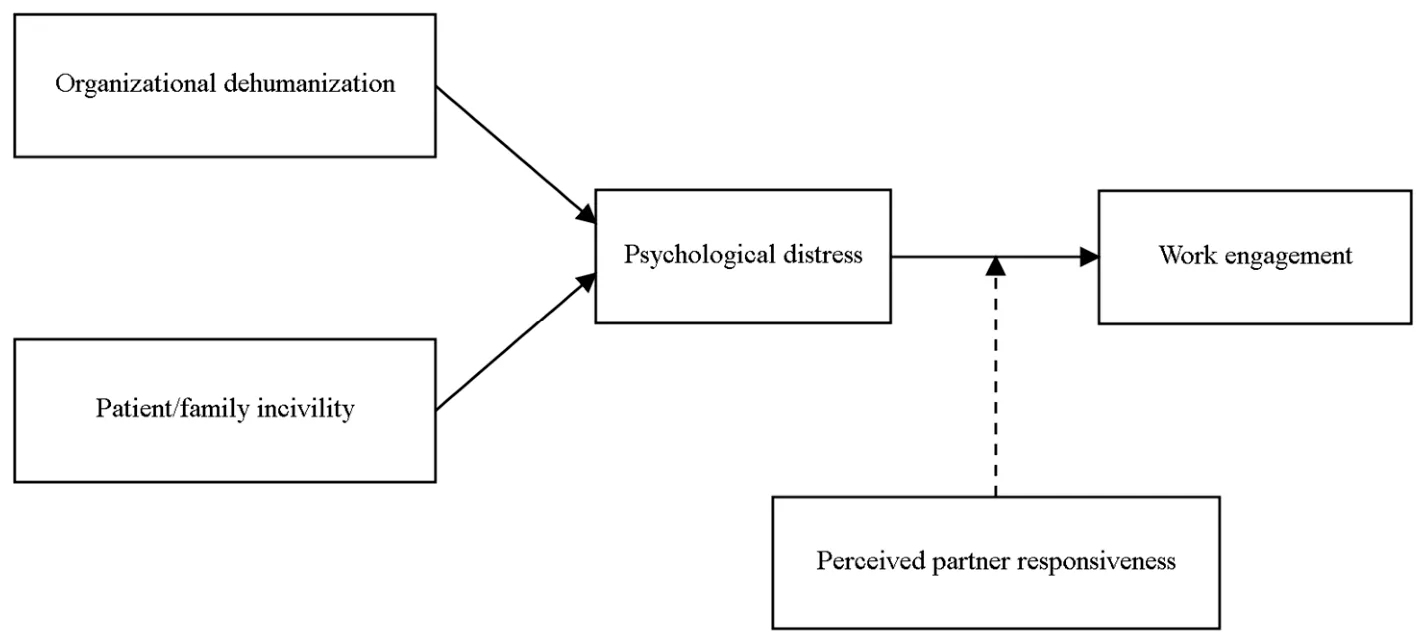
Proposed theoretical model. Solid arrows indicate hypothesized associations; the dashed arrow indicates moderation of the distress–engagement association by perceived partner responsiveness.

Based on the preceding arguments, we propose the moderated mediation model shown in Figure 1 and test the following hypotheses:

**H1.**  *Organizational dehumanization is positively associated with psychological distress.*

**H2.**  *Patient/family incivility is positively associated with psychological distress.*

**H3.**  *Psychological distress is negatively associated with work engagement.*

**H4.**  *Psychological distress mediates the associations of organizational dehumanization and patient/family incivility with work engagement.*

**H5.**  *Perceived partner responsiveness moderates the association between psychological distress and work engagement, such that the association is weaker when perceived partner responsiveness is higher.*

**H6.**  *The indirect associations of organizational dehumanization and patient/family incivility with work engagement through psychological distress are weaker when perceived partner responsiveness is higher.*

## 2. Materials and Methods

### 2.1. Participants and Procedure

Participants were recruited through Credamo, an online participant-recruitment platform. Eligibility was limited to married nurses. Survey access was restricted to respondents who identified nursing as their occupation. As an additional identity check, participants reported their professional title and were instructed to enter only the last four digits of their nurse practice certificate number; full certificate numbers were not requested. Participants received RMB 2 for each survey wave they completed. Responses across the three waves were matched using unique platform-assigned IDs.

Among the 477 valid Time 1 respondents, 391 completed Time 2 (82.0%), and 347 provided matched, usable data at Time 3 (88.7% of the Time 2 sample and 72.7% of the Time 1 sample). Excluding 22 participants who failed the attention check yielded a final analytic sample of 325 married nurses (68.1% of the Time 1 sample). Attrition analyses yielded two unadjusted differences. Nurses retained at Time 2 reported higher baseline patient/family incivility than those who did not continue after Time 1 (*p* = 0.035, d = 0.235), whereas Time 3 completers reported lower baseline organizational dehumanization than those who discontinued after Time 2 (*p* = 0.046, d = −0.365). Neither difference remained statistically significant after Holm correction; however, modest selective attrition cannot be excluded.

The study was approved by the Institutional Review Board of the Faculty of Psychology at Southwest University on 27 March 2025 (Approval No. H25080). All participants provided informed consent before data collection.

### 2.2. Measures

Validated Chinese-language versions were used when available. Measures without validated Chinese versions were translated and back-translated in accordance with Brislin’s procedure [38]. Two bilingual researchers independently translated the items into Chinese and reconciled their translations. A third bilingual researcher then back-translated the agreed Chinese version into English. The research team reviewed discrepancies between the original and back-translated versions and finalized the wording before survey administration. Unless otherwise noted, higher scores reflected higher levels of the corresponding constructs.

#### 2.2.1. Organizational Dehumanization

Organizational dehumanization was assessed using the 11-item scale developed by Caesens et al. [39]. Participants rated each item on a seven-point Likert scale ranging from 1 (strongly disagree) to 7 (strongly agree). A sample item was “My organization considers me as a tool to use for its own ends.” The item scores were averaged to produce a composite score. Cronbach’s alpha was 0.956.

#### 2.2.2. Patient/Family Incivility

Patient/family incivility was assessed using a four-item measure adapted from Grandey et al. [40]. Participants indicated how often patients or family members had argued with, shouted at, spoken rudely to, or insulted them or their team. Items were rated on a five-point frequency scale ranging from 1 (never) to 5 (always), and item scores were averaged to produce a composite score. Cronbach’s alpha was 0.784.

#### 2.2.3. Psychological Distress

Psychological distress was measured using the six-item Kessler Psychological Distress Scale developed by Kessler et al. [41]. To match the one-week interval between survey waves, participants indicated how often they had experienced each symptom during the past week using a five-point frequency scale ranging from 1 (never) to 5 (always). A sample item was “During the past week, how often did you feel that everything was an effort?” Item scores were averaged to produce a composite score. Cronbach’s alpha was 0.811.

#### 2.2.4. Perceived Partner Responsiveness

Perceived partner responsiveness was assessed using the eight-item responsiveness subscale developed by Crasta et al. [42], which measures the extent to which individuals feel understood, validated, and cared for by their partner. Participants rated each item on a six-point Likert-type scale. A sample item was “My partner really listens to me.” Item scores were averaged to produce a composite score. Cronbach’s alpha was 0.873.

#### 2.2.5. Work Engagement

Work engagement was assessed using the 18-item Job Engagement Scale developed by Rich et al. [1]. The scale assesses the physical, emotional, and cognitive energy individuals invest in their work. Participants rated each item on a five-point Likert-type scale ranging from 1 (strongly disagree) to 5 (strongly agree). A sample item was “I am enthusiastic in my job.” The 18 items were averaged to produce an overall work engagement score. Cronbach’s alpha was 0.878.

#### 2.2.6. Control Variables

Gender, age, education, years of nursing experience, and professional title were included as covariates in all PROCESS analyses. Education and professional title were dummy-coded, whereas age and years of nursing experience were treated as continuous variables. As a block, these covariates did not significantly increase the variance explained in psychological distress (Δ*R*^2^ = 0.015, Δ*F*(10, 312) = 0.80, *p* = 0.631) or work engagement (Δ*R*^2^ = 0.025, Δ*F*(10, 309) = 1.59, *p* = 0.110), but they were retained for consistent adjustment.

### 2.3. Data Analysis

Confirmatory factor analyses were conducted in R 4.4.0 using lavaan 0.7–2 with maximum-likelihood estimation. The hypothesized five-factor model was compared with alternative models, and discriminant validity was assessed using item-level heterotrait–monotrait ratios [43]. Common method variance was further examined using an orthogonal latent method factor [44].

Descriptive statistics, Cronbach’s alpha coefficients, and Pearson correlations were calculated in SPSS 27.0. Moderated mediation was tested using PROCESS Model 14 with 5000 bootstrap samples. Separate analyses were conducted for each workplace stressor, with the other stressor entered as a covariate. All models also adjusted for gender, age, education, years of nursing experience, and professional title. Psychological distress and perceived partner responsiveness were mean-centered, and conditional associations were estimated at the moderator mean and ±1 *SD*. Two-tailed tests used α = 0.05; bootstrap estimates were significant when their 95% confidence intervals excluded zero. *B*, SE, *β*, 95% confidence intervals, *R*^2^, Δ*R*^2^, and Cohen’s *f*^2^ were reported.

## 3. Results

### 3.1. Measurement Model and Common Method Bias

Although the three-wave design temporally separated the focal measures, all data were self-reported; additional diagnostic analyses were therefore conducted. The hypothesized five-factor model fit the data adequately, χ^2^(1024) = 1651.38, χ^2^/df = 1.61, CFI = 0.914, TLI = 0.910, RMSEA = 0.043, and SRMR = 0.050, and outperformed all alternative specifications (Table 1). These included the four-factor model combining organizational dehumanization with patient/family incivility, Δχ^2^(4) = 210.27, *p* < 0.001; the three-factor model combining both stressors with psychological distress, Δχ^2^(7) = 509.66, *p* < 0.001; and the one-factor model, Δχ^2^(10) = 1942.77, *p* < 0.001. All indicators loaded significantly on their intended factors (*p* < 0.001). HTMT values ranged from 0.361 to 0.665, with a value of 0.615 between organizational dehumanization and patient/family incivility; all were below the 0.85 criterion. Together, these findings supported the proposed factor structure and the empirical distinctiveness of the five constructs.

To assess residual common method variance, a freely estimated orthogonal common latent method factor was added to the five-factor model. The expanded model fit the data well, χ^2^(977) = 1383.08, CFI = 0.945, TLI = 0.939, RMSEA = 0.036, and SRMR = 0.041. Although the method factor accounted for an average of 3.4% of item variance, all trait loadings remained significant, and the latent correlations changed minimally relative to the trait-only model (maximum absolute change = 0.036). Thus, common method variance cannot be ruled out but is unlikely to be the primary explanation for the observed associations.

### 3.2. Descriptive Statistics

Table 2 summarizes the descriptive statistics and zero-order correlations. The two workplace stressors were moderately correlated, *r* = 0.534, *p* < 0.001. Both were associated with greater psychological distress (organizational dehumanization: *r* = 0.583; patient/family incivility: *r* = 0.506) and lower work engagement (organizational dehumanization: *r* = −0.452; patient/family incivility: *r* = −0.330), all *ps* < 0.001. Greater psychological distress was associated with lower work engagement, *r* = −0.567, *p* < 0.001, whereas higher perceived partner responsiveness was associated with lower psychological distress, *r* = −0.480, and greater work engagement, *r* = 0.563 (both *ps* < 0.001).

### 3.3. Testing the Hypothesized Moderated Mediation Model

In the covariate-adjusted first-stage regression (Table 3), the model explained 40.8% of the variance in psychological distress, *R^2^* = 0.408, *F*(12, 312) = 17.90, *p* < 0.001. Organizational dehumanization was positively associated with psychological distress, *B* = 0.160, SE = 0.019, *β* = 0.438, *t* = 8.384, *p* < 0.001, 95% CI [0.123, 0.198], supporting H1. Patient/family incivility also showed an independent positive association with psychological distress, *B* = 0.216, SE = 0.042, *β* = 0.271, *t* = 5.171, *p* < 0.001, 95% CI [0.134, 0.298], supporting H2.

The second-stage model retained both workplace stressors and included mean-centered psychological distress, mean-centered perceived partner responsiveness, and their interaction, together with the five covariates. As shown in Table 4, the model accounted for 52.2% of the variance in work engagement, *R^2^* = 0.522, *F*(15, 309) = 22.49, *p* < 0.001.

At the sample mean of perceived partner responsiveness, psychological distress was negatively associated with work engagement, *B* = −0.180, SE = 0.041, *β* = −0.246, *t* = −4.443, *p* < 0.001, 95% CI [−0.260, −0.101], supporting H3. At the sample mean of psychological distress, perceived partner responsiveness was positively associated with work engagement, *B* = 0.129, SE = 0.028, *β* = 0.243, *t* = 4.612, *p* < 0.001, 95% CI [0.074, 0.185].

More importantly, the psychological distress × perceived partner responsiveness interaction was positive and significant, *B* = 0.201, SE = 0.032, *β* = 0.305, *t* = 6.357, *p* < 0.001, 95% CI [0.139, 0.263]. The positive interaction indicated that the negative distress–engagement association weakened as perceived partner responsiveness increased. Adding the interaction explained an additional 6.3% of the variance, Δ*R^2^* = 0.063, Δ*F*(1, 309) = 40.41, *p* < 0.001, Cohen’s *f^2^* = 0.131, representing a small-to-moderate incremental effect.

After adjustment for both workplace stressors, the five covariates, psychological distress, perceived partner responsiveness, and their interaction, organizational dehumanization retained a small negative direct association with work engagement, *B* = −0.030, SE = 0.015, *β* = −0.110, *t* = −2.008, *p* = 0.046, 95% CI [−0.059, −0.001]. Patient/family incivility, however, was not directly associated with work engagement, *B* = −0.009, SE = 0.029, *β* = −0.015, *t* = −0.307, *p* = 0.759, 95% CI [−0.066, 0.048].

### 3.4. Simple Slope Analysis

**Figure 2:**
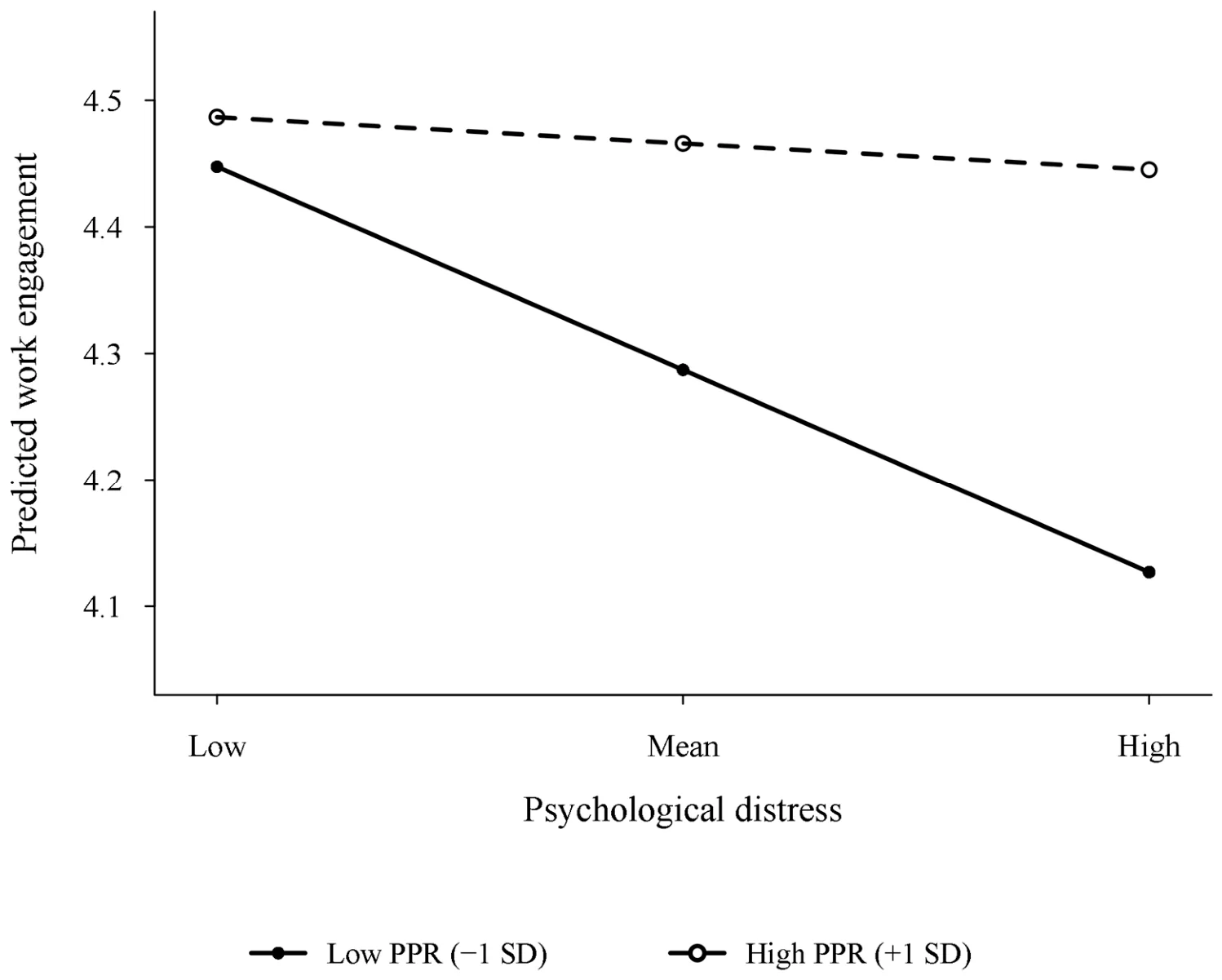
Conditional Association Between Psychological Distress and Work Engagement at Different Levels of Perceived Partner Responsiveness.

Simple slope analyses were conducted at one standard deviation below the mean, the mean, and one standard deviation above the mean of perceived partner responsiveness. Psychological distress was negatively associated with work engagement when perceived partner responsiveness was low, *B* = −0.319, SE = 0.041, *β* = −0.435, *t* = −7.875, *p* < 0.001, 95% CI [−0.399, −0.240], and at the mean, *B* = −0.180, SE = 0.041, *β* = −0.246, *t* = −4.443, *p* < 0.001, 95% CI [−0.260, −0.101]. At high responsiveness, the association was weaker and not statistically significant, *B* = −0.041, SE = 0.051, *β* = −0.056, *t* = −0.811, *p* = 0.418, 95% CI [−0.142, 0.059]. As Figure 2 shows, the negative distress–engagement association weakened as perceived partner responsiveness increased, supporting H5.

### 3.5. Conditional Indirect Associations

Conditional indirect associations are presented in Table 5. Organizational dehumanization was indirectly associated with lower work engagement through psychological distress at low perceived partner responsiveness, estimate = −0.051, bootstrap 95% CI [−0.075, −0.026], and at the mean, estimate = −0.029, bootstrap 95% CI [−0.047, −0.015]. At high responsiveness, the confidence interval included zero, estimate = −0.007, bootstrap 95% CI [−0.032, 0.012].

Patient/family incivility showed the same pattern. Its indirect association was significant at low perceived partner responsiveness, estimate = −0.069, bootstrap 95% CI [−0.120, −0.024], and at the mean, estimate = −0.039, bootstrap 95% CI [−0.069, −0.016]. At high responsiveness, the confidence interval included zero, estimate = −0.009, bootstrap 95% CI [−0.039, 0.019]. The significant indirect associations at the mean level of perceived partner responsiveness supported H4; whether these associations differed across levels of responsiveness was examined in the test of H6.

The indices of moderated mediation excluded zero for both organizational dehumanization, index = 0.032, bootstrap 95% CI [0.003, 0.052], and patient/family incivility, index = 0.043, bootstrap 95% CI [0.003, 0.086]. The corresponding high–low contrasts were also significant: organizational dehumanization, contrast = 0.045, bootstrap 95% CI [0.004, 0.072], and patient/family incivility, contrast = 0.060, bootstrap 95% CI [0.004, 0.119] (Table 6). Thus, the conditional indirect associations were significantly less negative at high than at low perceived partner responsiveness, supporting H6.

## 4. Discussion

This study examined how organizational dehumanization and patient/family incivility were associated with married nurses’ work engagement through psychological distress and whether perceived partner responsiveness conditioned this pattern. After covariate adjustment, both workplace stressors were independently associated with greater psychological distress, which was associated with lower subsequent work engagement. The distress–engagement association and the corresponding indirect associations were weaker at higher levels of perceived partner responsiveness. Organizational dehumanization also retained a small direct association with work engagement, whereas patient/family incivility did not.

The findings sharpen the distinction between organizational dehumanization and patient/family incivility as sources of nurses’ occupational strain. Organizational dehumanization reflects devaluation by the employing institution and may erode nurses’ dignity, professional worth, and agency, whereas patient/family incivility occurs during care encounters and may place more immediate demands on emotional regulation. Previous nursing research has linked organizational dehumanization to work stress and lower engagement [15] and patient/family incivility to burnout [21] but has generally examined these stressors separately. By modeling both simultaneously, the present study showed that each retained a unique association with psychological distress after their shared variance and the covariates were accounted for, supporting their conceptualization as related but distinct workplace demands. Organizational dehumanization also retained a small direct association with engagement after distress was included, whereas patient/family incivility did not. Within the present model, this pattern suggests that distress accounted for more of the association between patient/family incivility and engagement, while organizational dehumanization may also relate to engagement through unmeasured processes. This interpretation remains tentative, however, because the difference in statistical significance does not itself establish different underlying mechanisms.

These findings add source and stage specificity to broad demand–resource accounts. JD–R and COR theories explain how demanding conditions generate strain by consuming or threatening valued resources but say less about whether demands from different workplace sources retain unique associations with the same strain response or when resources outside work enter this process [27,29]. In the present study, organization-based dehumanization and care-recipient incivility were empirically distinct yet converged on psychological distress. The moderating association of perceived partner responsiveness emerged at the later distress–engagement stage, where higher responsiveness was associated with a weaker negative relationship between distress and subsequent engagement. Consistent with the work–home resources model [30], this pattern integrates demand source, resource domain, and the stage at which a home-domain resource may become relevant within the same strain process.

The moderation findings help locate perceived partner responsiveness within the proposed strain process. Because partners cannot directly alter nurses’ exposure to organizational dehumanization or patient/family incivility, responsiveness is more likely to become relevant after distress has emerged, when nurses are processing the emotional residue of difficult work experiences. Although perceived partner responsiveness was inversely related to psychological distress, this zero-order association is conceptually distinct from the moderation tested here: whether the relationship between distress and subsequent work engagement varies across levels of responsiveness. Feeling understood, validated, and cared for may help preserve the psychological capacity needed for subsequent work engagement. This experienced quality of a partner’s response, rather than the mere availability of support, is central to perceived partner responsiveness [35]. Such an interpretation is consistent with longitudinal evidence linking responsiveness to greater well-being and lower affective reactivity [36] and with the work–home resources model [30]. Empirically, both the distress–engagement association and the corresponding indirect associations were weaker at higher levels of responsiveness. Together with the significant interaction and high–low contrasts, the nonsignificant conditional estimates at high responsiveness support an interpretation of attenuation rather than elimination; they do not demonstrate the absence of adverse associations. Because supportive interactions and recovery experiences were not directly measured, partner-facilitated recovery remains a theoretically plausible rather than empirically established mechanism.

The restriction on married nurses defines the scope of the findings rather than implying that marriage is inherently protective. Marriage provided a common relational context and an identifiable partner referent, whereas the resource of theoretical interest was perceived partner responsiveness rather than marital status itself. From a work–home resources perspective, recurring interactions with a spouse may provide opportunities for emotional support and recovery [30], but marriage alone does not ensure that these interactions are beneficial. Their value depends on whether the spouse is experienced as understanding, validating, and caring [35]. Accordingly, a low-responsiveness marriage may offer little benefit, whereas nurses in cohabiting or other committed relationships may receive a functionally similar resource from a responsive partner. Nurses without intimate partners may instead rely on family members, friends, coworkers, or supervisors, although support originating from different relationships and life domains may not operate identically. The present findings should therefore be interpreted as reflecting variation in perceived spousal responsiveness among married nurses, not as evidence that marriage itself promotes recovery or engagement.

Practical implications begin with preventing avoidable strain at its organizational source. When organizational practices contribute to psychological distress, hospitals should not frame diminished engagement mainly as a failure of individual resilience. Reducing dehumanizing treatment requires management practices that affirm nurses’ dignity, voice, and professional agency, including respectful communication, transparent explanations of decisions and constraints, meaningful participation in scheduling and workflow design, and recognition of clinical judgment. These efforts should be supported by effective workload governance: persistent understaffing, uneven assignments, excessive overtime, and consecutive shifts should trigger adjustments in staffing, rosters, or resource allocation rather than become normalized. Counseling and resilience programs may complement these structural measures but should not substitute for them. Because no intervention was tested, these recommendations represent priorities for organizational action and evaluation rather than effects demonstrated by the present study.

Patient/family incivility should be managed as an organizational safety and care-environment issue rather than as an interpersonal burden for individual nurses to absorb. Prevention requires reducing avoidable interactional friction while establishing clear boundaries for acceptable conduct. Timely explanations of care processes, waiting times, professional roles, and treatment progress, together with opportunities for questions and appropriate participation in care decisions, may reduce misunderstandings and foster a more respectful patient–provider relationship. Hospitals should complement these communication practices with explicit standards for patient and visitor conduct and accessible channels for expressing concerns. When behavioral boundaries are crossed, nurses need nonpunitive reporting procedures, prompt supervisory follow-up, de-escalation protocols, and post-incident support. Evidence from structured workplace-civility interventions indicates that coordinated organizational action can improve the social climate of healthcare workplaces [45], although its effectiveness in reducing incivility from patients and families requires direct evaluation. Efforts to improve mutual understanding should therefore reinforce formal institutional protections, not transfer responsibility for preventing mistreatment to individual nurses.

Beyond reducing avoidable demands, hospitals should protect the conditions needed for recovery. Work design and supervisory practices partly determine whether nurses have sufficient time and support to recover; recovery should therefore not be treated as a private responsibility borne by nurses and their families. Predictable schedules, adequate rest between shifts, and limits on nonessential after-hours contact can safeguard recovery time, while confidential employee assistance programs and accessible psychological services can provide support when distress persists. Supervisors can reinforce these conditions by respecting nonwork boundaries and responding constructively to work–family needs. Field evidence suggests that family-supportive supervisor training can strengthen perceived support and improve employee outcomes, although its benefits may vary with employees’ family-to-work conflict [46]. Perceived partner responsiveness is best understood as a complementary relational resource: it may aid recovery from unavoidable demands but cannot offset dehumanizing organizational practices or recurrent patient/family incivility. Organizations can facilitate recovery and offer voluntary support but should not intervene in employees’ intimate relationships. An effective occupational mental health strategy should therefore integrate the prevention of avoidable strain, protection of recovery time, and timely professional support.

Cultural context may shape how partner responsiveness is conveyed, recognized, and related to well-being. East Asians report lower perceived responsiveness than European Americans, partly reflecting culturally patterned differences in self-consistency [47], while relational concerns may make Asian and Asian American adults less likely to seek explicit support [48]. The same supportive behavior may also convey different relational meanings across cultural groups [49]. Yet perceived partner responsiveness has been positively associated with well-being in both the United States and Japan, although more strongly in the United States [50]. Responsiveness may therefore be broadly relevant without being culturally uniform in its expression or strength. In the present Chinese sample, higher perceived partner responsiveness was associated with a weaker negative relationship between distress and subsequent work engagement. However, because the study neither measured cultural orientations and support practices nor included a cross-cultural comparison, it cannot determine whether this pattern is culturally specific or generalizable across contexts.

### 4.1. Limitations

Several limitations should be considered when interpreting these findings. First, although the three-wave design separated the focal variables in time, all measures were self-reported and the study remained observational. The measurement-model and common-method-factor analyses indicated that common method variance was unlikely to account fully for the observed associations, but residual same-source bias cannot be excluded [44]. The one-week intervals were chosen to align with the past-week reference period of the psychological distress measure and to provide temporal separation for examining short-term associations. Nevertheless, this interval represents a methodological choice rather than an empirically established timescale for the broader stress–engagement process. Whether the observed associations persist over shorter or longer intervals remains unknown. Temporal ordering, however, does not establish causality; the conditional indirect associations may also reflect reciprocal relationships or unmeasured confounding and should not be interpreted as evidence of causal mediation. Finally, retention analyses revealed modest differences across waves, none of which remained significant after Holm correction. Selective attrition therefore remains possible, although the direction and magnitude of any resulting bias are uncertain.

Second, participants were recruited from a nonprobability online panel of married nurses in China, which limits both sample representativeness and the scope of generalization. Occupational screening and professional information helped verify eligibility but did not make the sample representative of the broader nursing workforce. The findings also capture variation in perceived spousal responsiveness within marriage, not the effect of marital status itself; they therefore provide no evidence that marriage is inherently protective. Nurses in cohabiting or other relationship contexts may have access to functionally comparable relational resources, while cultural norms may shape how responsiveness is expressed, interpreted, and related to work outcomes [47,50]. Because the sample was not designed to represent nurses across regions or healthcare systems, the findings should be generalized cautiously beyond the relationship, occupational, and cultural contexts examined here. Replication in diverse regions and healthcare systems is needed to assess their broader applicability.

Third, the model necessarily represents only part of the broader system of demands and resources shaping nurses’ distress and engagement. Other occupational conditions, such as staffing, workload, shift arrangements, ward characteristics, and organizational justice, as well as unmeasured workplace and personal resources, may have contributed to the observed associations. Perceived partner responsiveness is appropriately assessed as a subjective relational experience; however, nurses’ reports alone cannot identify the partner behaviors or interactions underlying that experience. Nor did the study directly assess supportive exchanges or recovery experiences. The moderation finding therefore locates where perceived responsiveness was associated with attenuation in the proposed strain process but does not establish how that attenuation occurred.

### 4.2. Implications for Future Research

Advancing this work requires designs that clarify temporal direction and observe recovery more directly. Longer panel studies with repeated assessments could test reciprocal relationships and determine whether the proposed pattern persists over time, whereas shift-level diary studies could trace how workplace stressors carry into nonwork recovery and engagement during subsequent shifts. Multisource data—including supervisor or peer ratings and administrative indicators of staffing, scheduling, and absence—would reduce reliance on nurses’ reports alone. Partner reports should complement rather than replace those reports because responsiveness is defined partly by how partner behavior is experienced. Dyadic diaries could further connect enacted support with perceived responsiveness, recovery experiences, and next-shift engagement. Finally, experimental or quasi-experimental studies targeting workplace demands or recovery resources could provide stronger evidence about whether changing these conditions reduces distress and sustains engagement.

Generalizability should be examined in terms of what relational resources do, rather than whether they occur within marriage. Comparing married, cohabiting, and unpartnered nurses could show whether responsiveness from an intimate partner and support from family members, friends, coworkers, or supervisors provide comparable recovery benefits or operate through different processes. Cross-cultural comparisons should first establish measurement invariance and assess individual cultural orientations rather than equate country with culture. They should also distinguish explicit verbal reassurance from practical help, quiet companionship, and sensitivity to unspoken needs. Such comparisons would clarify whether cultural variation lies in how responsive support is expressed and recognized, or in the strength of its association with recovery and work engagement.

Future research should develop a more differentiated account of how resources from different domains enter the strain process. Coworker support, supervisor support, family-supportive supervisor behavior, home-domain partner responsiveness, personal resources such as resilience and mindfulness, and recovery experiences such as psychological detachment and relaxation may not serve the same function. Comparative studies should examine whether these resources reduce exposure or reactivity to workplace demands, limit the development of psychological distress, or facilitate recovery after distress has emerged, as well as whether they complement or substitute for one another. Although reviews of mindfulness- and resilience-focused interventions among nurses are encouraging, the evidence remains insufficiently consistent to establish when, for whom, and through which processes these approaches are effective [51,52]. Qualitative interviews with nurses and their partners or other close supporters could identify the responses experienced as helpful after demanding shifts and guide the development of more precise dyadic measures and targeted interventions.

## 5. Conclusions

In this three-wave study of 325 married nurses in China, organizational dehumanization and patient/family incivility were independently associated with greater psychological distress at the subsequent wave; greater distress was, in turn, associated with lower work engagement at the following wave. Both the distress–engagement association and the corresponding indirect associations were weaker at higher levels of perceived partner responsiveness. Organizational dehumanization also retained a small direct association with engagement, whereas patient/family incivility did not. Together, these findings distinguish workplace demands by source and suggest that perceived partner responsiveness may be relevant as a family-domain relational resource at the later distress–engagement stage. Because the data were observational and self-reported, they do not establish causal mediation or partner-facilitated recovery. For nursing management, the broader implication is to pair prevention with recovery by reducing dehumanizing practices and patient/family incivility, maintaining manageable work demands, and protecting opportunities for recovery outside work. Partner and family support may complement these efforts but cannot substitute for organizational action. Prospective intervention studies are needed to evaluate these approaches.

## Figures and Tables

**Table 1:** Comparison of Alternative Measurement Models.

Model	χ^2^	df	χ^2^/df	CFI	TLI	RMSEA	SRMR	Δχ^2^(df)
Five-factor model	1651.38	1024	1.61	0.914	0.91	0.043	0.050	—
Four-factor: OD + PFI combined	1861.64	1028	1.81	0.886	0.88	0.050	0.056	210.27(4) ***
Three-factor: OD + PFI + PD combined	2161.04	1031	2.10	0.846	0.838	0.058	0.068	509.66(7) ***
One-factor model	3594.14	1034	3.48	0.651	0.635	0.087	0.099	1942.77(10) ***

Note. *N* = 325. OD = organizational dehumanization; PFI = patient/family incivility; PD = psychological distress. *** *p* < 0.001.

**Table 2:** Descriptive Statistics and Correlations Among Variables.

Variable	M	*SD*	1	2	3	4	5
1. Organizational dehumanization	2.84	1.37	-				
2. Patient/family incivility	2.10	0.63	0.534 **	-			
3. Psychological distress	1.70	0.50	0.583 **	0.506 **	-		
4. Perceived partner responsiveness	4.90	0.69	−0.481 **	−0.300 **	−0.480 **	-	
5. Work engagement	4.34	0.37	−0.452 **	−0.330 **	−0.567 **	0.563 **	-

Note. *N* = 325. ** *p* < 0.01 (two-tailed).

**Table 3:** First-Stage Regression Model Predicting Psychological Distress.

Predictor	*B*	SE	*β*	*t*	*p*	95% CI for *B*
Organizational dehumanization	0.160	0.019	0.438	8.384	<0.001	[0.123, 0.198]
Patient/family incivility	0.216	0.042	0.271	5.171	<0.001	[0.134, 0.298]

Note. *N* = 325. Covariates were gender, age, education, years of nursing experience, and professional title. *B* = unstandardized coefficient; *β* = standardized coefficient; CI = confidence interval.

**Table 4:** Second-Stage Model Predicting Work Engagement.

Predictor	*B*	SE	*β*	t	*p*	95% CI for *B*
Organizational dehumanization	−0.030	0.015	−0.110	−2.008	0.046	[−0.059, −0.001]
Patient/family incivility	−0.009	0.029	−0.015	−0.307	0.759	[−0.066, 0.048]
Psychological distress	−0.180	0.041	−0.246	−4.443	<0.001	[−0.260, −0.101]
Perceived partner responsiveness	0.129	0.028	0.243	4.612	<0.001	[0.074, 0.185]
Psychological distress × perceived partner responsiveness	0.201	0.032	0.305	6.357	<0.001	[0.139, 0.263]

Note. *N* = 325. Covariates were gender, age, education, years of nursing experience, and professional title. *B* = unstandardized coefficient; *β* = standardized coefficient; CI = confidence interval.

**Table 5:** Conditional Indirect Associations Between Workplace Stressors and Work Engagement Through Psychological Distress.

Independent Variable	Perceived Partner Responsiveness	Estimate	Boot 95% CI
Organizational dehumanization	Low (−1 *SD*)	−0.051	[−0.075, −0.026]
	Mean	−0.029	[−0.047, −0.015]
	High (+1 *SD*)	−0.007	[−0.032, 0.012]
Patient/family incivility	Low (−1 *SD*)	−0.069	[−0.120, −0.024]
	Mean	−0.039	[−0.069, −0.016]
	High (+1 *SD*)	−0.009	[−0.039, 0.019]

Note. *N* = 325. Estimates are unstandardized. Models adjusted for the other workplace stressor and the five covariates. Bootstrap 95% CIs were based on 5000 resamples.

**Table 6:** High–Low Contrasts of Conditional Indirect Associations.

Independent Variable	High–Low Contrast	Boot 95% CI
Organizational dehumanization	0.045	[0.004, 0.072]
Patient/family incivility	0.060	[0.004, 0.119]

Note. *N* = 325. Contrasts were calculated as high minus low. Bootstrap 95% CIs were based on 5000 resamples. Models adjusted for the other workplace stressor and the five covariates.

## Data Availability

The data that support the findings of this study are available from the corresponding author upon reasonable request. The data are not publicly available due to privacy and ethical restrictions.
